# Persistent excess mortality in post-pandemic Japan: current evidence, methodological limitations, and future directions

**DOI:** 10.3389/fpubh.2026.1878166

**Published:** 2026-06-23

**Authors:** Hiroshi Kusunoki, Ryota Sakai, Yumi Watanabe, Hideki Kakeya

**Affiliations:** 1 Department of Internal Medicine, Osaka Dental University, Hirakata, Japan; 2 Department of Rheumatology and Clinical Immunology, Saitama Medical Center, Saitama Medical University, Kawagoe, Japan; 3 Division of Preventive Medicine, Niigata University Graduate School of Medical and Dental Sciences, Niigata, Japan; 4 Institute of Systems and Information Engineering, University of Tsukuba, Tsukuba, Japan; 5 Transdisciplinary Review Association, Moriya, Japan

**Keywords:** all-cause mortality, booster vaccination, COVID-19, excess mortality, healthy vaccinee bias, individual-level data infrastructure, methodological limitations

## Abstract

More than six years after the emergence of COVID-19, Japan continues to experience persistently elevated excess mortality despite entering a post-pandemic phase. Excess mortality, defined as the difference between observed and expected deaths, reflects both direct and indirect effects of the pandemic, including healthcare disruption, delayed care, and broader social changes. In Japan, mortality decreased slightly in 2020 but increased markedly from 2022 onward, with annual excess deaths approaching 100,000, despite widespread vaccination and the predominance of the less virulent Omicron variant. Multiple factors may underlie this sustained increase, including population aging, changes in healthcare access, delayed diagnosis and treatment, regional disparities, and psychosocial stress. Some ecological and observational studies have reported a statistical association between repeated COVID-19 booster vaccination and excess mortality in Japan; however, these findings do not establish causality, and a causal relationship cannot currently be concluded. Against this background, this narrative review examines persistent excess mortality in Japan, uses the literature on repeated booster vaccination as an illustrative example to highlight methodological limitations, and discusses the need for nationwide, linked individual-level data infrastructure and appropriate analytical frameworks for more robust future evaluations.

## Introduction

More than six years after the emergence of coronavirus disease 2019 (COVID-19) and three years after its reclassification as a Category 5 infectious disease under Japan’s Infectious Disease Control Law, excess mortality in Japan remains persistently elevated. Excess mortality, defined as the difference between observed and expected deaths, represents a comprehensive indicator of the societal impact of a pandemic, encompassing not only the direct effects of infection but also indirect consequences such as healthcare disruption, delayed medical care, and changes in social behavior. Although many countries experienced increased excess mortality during 2020–2021 followed by subsequent variation, Japan remains notable for sustained elevation even in the post-acute phase (1).

This persistent excess mortality constitutes an important public health concern and has prompted debate regarding its underlying causes. Some ecological and observational studies have reported statistical associations between repeated COVID-19 booster vaccination and excess mortality; however, these findings do not establish causality, and the current evidence remains insufficient for causal inference because of substantial methodological limitations and potential confounding (2).

In contrast, international studies have consistently demonstrated that COVID-19 booster vaccination reduces hospitalization, severe disease, and COVID-19–related mortality, particularly among older and high-risk populations. Studies from the United States, the United Kingdom, France, and Sweden have reported lower risks of severe COVID-19 outcomes and, in some analyses, reduced all-cause mortality among vaccinated individuals, without evidence supporting broad harmful effects of booster vaccination (3–8). Nevertheless, extrapolation of these findings to Japan should be approached cautiously, as differences in population aging, cumulative vaccine exposure, dosing intervals, and healthcare systems may influence both the magnitude and direction of observed effects. Furthermore, protection against COVID-19–related mortality is not equivalent to effects on all-cause or excess mortality.

Direct evidence from Japan remains limited, particularly regarding large-scale nationwide individual-level analyses evaluating mortality according to cumulative booster dose. Existing Japanese real-world data (VENUS) have not demonstrated an increased risk of death associated with mRNA booster vaccination (9), but more comprehensive population-level analyses are still lacking. While the VENUS study included a substantial sample size among older adults, its limited number of cases in the younger age group (18–64 years) may have resulted in insufficient statistical power. Moreover, the persistence of markedly low odds ratios despite adjustment for underlying comorbidities suggests the potential presence of residual healthy vaccinee bias, whereby individuals in better overall health may have been more likely to undergo vaccination. The follow-up period was also confined to a very short post-vaccination window of 21–42 days. Therefore, although the study may provide reasonably strong support for the short-term safety profile of vaccination, particularly with respect to the absence of increased mortality risk, greater caution is required in interpreting the reported effectiveness for substantially reducing all-cause mortality, given the possibility of bias-related overestimation.

Against this background, the present article reviews Japanese evidence regarding repeated COVID-19 booster vaccination and excess mortality while highlighting the need for more robust epidemiological data. Specifically, this study aims to characterize temporal trends in excess mortality in Japan, clarify the methodological limitations inherent in their interpretation, and discuss the data infrastructure required to enable more rigorous vaccine-related analyses.

This narrative review aims to characterize persistent excess mortality in post-pandemic Japan, summarize current evidence, and clarify the methodological limitations of current approaches to causal inference. Studies of repeated COVID-19 booster vaccination are discussed solely as an illustrative example of the broader methodological challenges arising from Japan’s current data environment, rather than as a central conclusion of the paper. The review highlights the need for a robust nationwide individual-level data infrastructure and appropriate causal frameworks for future evaluation.

### Trends in excess mortality in Japan before and during the COVID-19 pandemic

In evaluating excess mortality, it is necessary to estimate expected deaths based on pre-pandemic mortality trends. Below, mortality patterns from 2015 to 2019 are used as the baseline to examine changes after the onset of the COVID-19 pandemic.

In Japan, the total number of deaths temporarily declined in 2020 but increased from 2021 onward, remaining at high levels in 2022 and beyond. High mortality levels have also been suggested for 2024 and 2025, indicating that this phenomenon is not a single-year fluctuation but should be interpreted as sustained excess mortality. However, this temporal pattern alone does not allow causal attribution to specific factors. It is necessary to distinguish between baseline increases in mortality associated with population aging and additional changes occurring after the pandemic.

**Figure 1:**
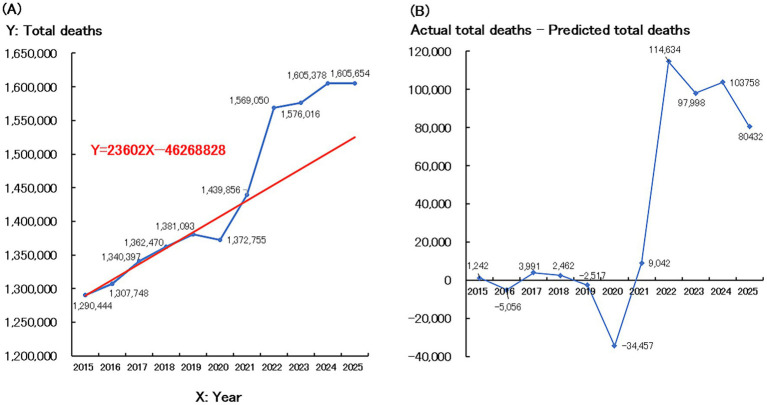
**(A)** Trends in the annual number of deaths in Japan since 2015. The x-axis represents calendar year. The regression equation and corresponding linear regression line for the pre-pandemic period (2015–2019) are shown in red. **(B)** Trends in the annual difference between observed and predicted total deaths (i.e., observed minus predicted). The figure is adapted from Reference 10, with the provisional 2025 annual number of deaths (Ref. 11) added.

Figure 1A presents annual all-cause mortality in Japan and is consistent with previously reported trends (10). Because mortality had been increasing approximately linearly before the COVID-19 pandemic, largely due to population aging, a linear regression model based on pre-pandemic data from 2015 to 2019 is used to define the expected baseline, as shown by the red line in Figure 1A. Figure 1B illustrates excess mortality estimated as the difference between observed deaths and model-predicted deaths from 2015 onward. National mortality decreased slightly in 2020, increased markedly in 2021 and 2022, remained elevated in 2023, and is projected to remain high in 2024, with excess deaths approaching 100,000. Preliminary national statistics indicate that total deaths in 2025 were similar to those in 2024 (11), suggesting that excess mortality estimated by the regression model may have plateaued or slightly decreased in 2025, although future trends remain uncertain.

Total mortality in Japan during the pre-pandemic period (2015–2019) increased in a remarkably linear manner, supporting the validity of estimating the expected mortality baseline using a linear regression model. Nevertheless, the use of unadjusted mortality counts has inherent limitations in the context of Japan’s rapidly aging population. Stronger evidence may be obtained through the application of more sophisticated counterfactual models, such as age-standardized mortality rates or interrupted time-series analyses incorporating multiple covariates.

An example of such an approach is provided by the following study. More detailed analyses incorporating adjustment for potential confounding factors have yielded broadly consistent findings. A study using Japanese vital statistics from 2015 to 2023 applied a two-stage interrupted time-series design comparing mortality during the pandemic period (2020–2023) with the pre-pandemic period (2015–2019), adjusting for seasonality, long-term trends, temperature, and influenza activity (12). Consistent with the present exploratory analysis, the study reported a slight decline in excess mortality in 2020, a reversal to increasing mortality in 2021, a marked increase in 2022, and persistently elevated mortality in 2023. Since the onset of the COVID-19 pandemic, life expectancy in Japan has stagnated, which may contribute to an acceleration of population decline (13). It is important to distinguish whether the observed increase in excess mortality is attributable solely to population ageing or to external factors such as the COVID-19 pandemic.

There have been concerns in Japan that the marked increase in excess mortality since 2022 may be associated with the initiation of COVID-19 vaccination in 2021. However, it should be emphasized that the findings presented here remain hypothesis-generating in nature.

### Observed associations between repeated COVID-19 vaccination and excess mortality in Japan

Several studies in Japan have examined the association between repeated COVID-19 booster vaccination and mortality. While these studies are useful for hypothesis generation, their interpretation requires careful consideration of methodological limitations inherent to observational and ecological study designs.

Japan implemented one of the most intensive COVID-19 booster vaccination programs globally, with repeated vaccination campaigns following the emergence of the Omicron variant (14). As a result, cumulative vaccine exposure became relatively high, particularly among older adults and high-risk populations. In this context, several studies have explored associations between vaccination intensity and excess mortality.

A recent preprint reported that age-adjusted excess mortality showed a negative correlation with the number of vaccine doses during the early pandemic phase (*r* = −0.36, *p* = 0.013), whereas a positive correlation was observed in the later phase when additional booster doses were introduced (*r* = 0.55, *p* = 0.000064) (15). However, these ecological associations are sensitive to temporal changes in infection dynamics, population structure, healthcare capacity, and other residual confounders, and therefore cannot distinguish causal effects.

**Figure 2:**
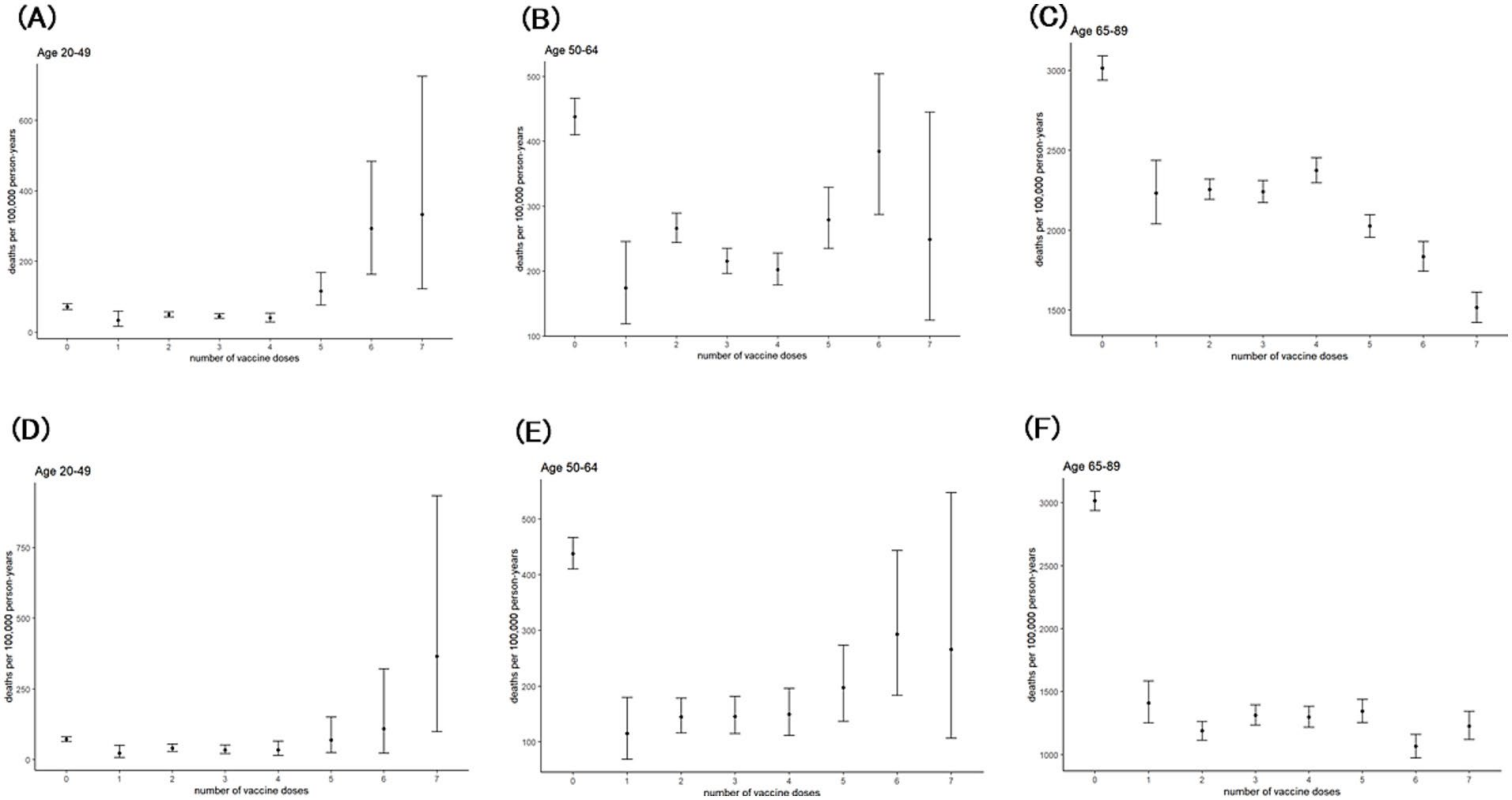
Unstandardized all-cause mortality rates by number of vaccine doses. Age groups: 20–49 **(A)**, 50–64 **(B)**, and 65–89 **(C)**. Error bars indicate 95% confidence intervals. The figures are adapted from Reference 16. Unstandardized all-cause mortality rates by number of vaccine doses, with a 90-day window after vaccination applied. Age groups: 20–49 **(D)**, 50–64 **(E)**, and 65–89 **(F)**. Error bars indicate 95% confidence intervals. The figures are adapted from Reference 17. Vaccination dose number was treated as a time-dependent covariate, and person-time was allocated dynamically according to changes in vaccination status. Mortality rate comparisons and confidence interval estimation were based on Poisson regression, which assumes equidispersion between the mean and variance. The study did not explicitly specify whether a post-vaccination lag or masking period (e.g., 14 days), during which individuals would remain classified under their previous dose category or be excluded from analysis, was implemented. Follow-up termination due to administrative censoring or relocation implicitly assumes non-informative right censoring, whereby censoring occurs independently of mortality risk. Furthermore, although analyses were stratified using broad age bands (e.g., 20–49 years), no within-stratum age standardization or adjustment was performed to address residual differences in age distribution.

The study by Iwamoto et al. (16), which utilized municipal registry data, reported differences in mortality patterns by cumulative vaccine dose. In Figures 2A–C show unstandardized all-cause mortality rates stratified by number of vaccine doses. Among individuals aged 65–89 years, mortality appears to decrease with increasing vaccine doses, superficially suggesting a protective effect of vaccination. However, this pattern may largely reflect the “healthy vaccinee bias,” whereby healthier individuals are more likely to receive repeated booster doses.

Figures 2D–F present mortality rates using a 90-day post-vaccination exclusion window (17). Although the overall patterns were broadly similar to those in Figures 2A–C,F showed that, among individuals aged 65–89 years, only the unvaccinated group exhibited clearly higher mortality, whereas mortality rates among those receiving 1–7 doses were relatively similar. Compared with the apparent gradient observed in Figure 2C, this attenuation after applying the 90-day window may indicate reduced influence of healthy vaccinee bias and could suggest that the incremental benefit of repeated booster vaccination on all-cause mortality is limited in this age group.

In contrast, among younger individuals aged 20–49 years in Figures 2A,D, higher cumulative vaccine doses were associated with higher observed all-cause mortality. This finding is likewise difficult to interpret causally and may instead reflect confounding, as individuals with underlying diseases or poorer baseline prognosis may have been more likely to receive repeated booster vaccination. However, because important confounders—such as comorbidities, prior infection history, and healthcare utilization—were not adjusted for, these findings should therefore be interpreted strictly as hypothesis-generating observations. Ecological correlations cannot disentangle vaccination effects from time-varying confounding, population structure, healthcare access, or infection dynamics and should not be regarded as evidence supporting either beneficial or harmful causal effects.

Such ecological associations are particularly vulnerable to regional heterogeneity and time-varying epidemiological conditions and do not establish individual-level causal relationships. Furthermore, although booster vaccination may have reduced severe COVID-19 outcomes, it did not clearly prevent increases in all-cause mortality among older populations with high booster uptake. Even if repeated booster vaccination substantially increased antibody titers, it remains uncertain whether repeated boosting had no effect on, contributed to, or helped mitigate increases in all-cause mortality. Clarification of these relationships will require further analyses using appropriately adjusted individual-level data. Accordingly, these findings should be interpreted not as causal evidence of vaccine effectiveness or safety, but as a signal requiring further validation.

At the prefectural level, Takahashi reported an increase in excess mortality after 2021, with associations observed with population aging, COVID-19 mortality, and regional disparities in healthcare access, particularly in rural and resource-limited areas. Notably, early vaccination phases were ecologically associated with lower excess mortality and later phases with higher excess mortality (18). Hypotheses such as immune imprinting and reduced vaccine effectiveness against emerging variants have been proposed; however, causal relations were not directly tested within these ecological frameworks (19).

In parallel, post-marketing surveillance and short-term safety evaluations conducted in Japan have not identified major new safety signals across a wide range of outcomes, although continued monitoring of specific adverse events remains warranted.

### Safety signals and adverse events following COVID-19 mRNA vaccination

In vaccine safety surveillance, “signal detection” is conducted using databases such as the Vaccine Adverse Event Reporting System (VAERS) to statistically identify whether there is a potential association between a vaccine and an adverse event. Harpaz et al. (20) pointed out that for products such as COVID-19 vaccines, where multiple vaccines are introduced rapidly and on a large scale, conventional pharmacovigilance methods alone may be insufficient to adequately detect safety signals. They proposed a more refined statistical approach and demonstrated herpes zoster and tinnitus as potential novel adverse events detectable by this method (20).

The following represent rare adverse events that have been recognized as having established or plausible associations with COVID-19 mRNA vaccination. Large-scale multinational collaborative analyses and post-marketing surveillance studies have identified several rare but established adverse events following COVID-19 mRNA vaccination—such as myocarditis, pericarditis, Guillain-Barré syndrome, and cerebral venous sinus thrombosis—but also other potential safety signals that require verification through additional research (21). International studies, including those in Canada, have not identified an increased short-term risk of events such as sudden cardiac death in young adults; however, residual confounding cannot be excluded, including differences in healthcare-seeking behavior (22). Overall, the safety profile is considered acceptable. However, some of the observed events may be clinically significant and warrant further investigation.

Within Japan, short-term safety has been evaluated using municipal administrative data analyzed with the self-controlled case series (SCCS) method, which compares risk and control periods within the same individual to assess associations between vaccination and adverse events. This analysis generally showed no increased risk for most evaluated outcomes, supporting the overall safety of COVID-19 mRNA vaccines. However, a potential signal for pulmonary embolism following the first dose was observed and warrants continued surveillance (23). In addition, expert review systems in Japan have not confirmed any deaths as causally related to vaccination, nor have they identified clear population-level mortality safety signals (24). Overall, while no major safety concerns have been established, selected signals continue to require careful monitoring.

In contrast, certain events proposed to be associated with COVID-19 mRNA vaccination, such as cancer-related outcomes, remain exploratory hypotheses. Observational and ecological studies have reported temporal associations between vaccination and outcomes such as cancer-related indicators; however, these do not establish causality (25, 26). Proposed biological mechanisms—such as effects of spike protein or immune modulation—remain hypothetical and insufficient to explain population-level mortality trends (2). Data from compensation systems, including higher numbers of recognized cases in Japan, are influenced by reporting and evaluation processes and cannot be directly interpreted as causal risk (27, 28). Overall, the relationship between repeated booster vaccination and all-cause or excess mortality remains unresolved.

Further evaluation requires robust, well-adjusted analyses based on large-scale, individual-level data with sufficient follow-up. Clear separation of evidence types—observational, descriptive, mechanistic, and surveillance—is essential to avoid misinterpretation. Strengthening nationwide data integration, improving transparency in safety monitoring, and maintaining continuous surveillance of rare adverse events will be critical for accurately assessing vaccine safety and informing evidence-based vaccination policy.

### Development of Nationwide data infrastructure for vaccine safety evaluation in Japan

In Japan, vaccination records, clinical information, and mortality data are not yet sufficiently integrated. As a result, rigorous evaluations with adequate confounder adjustment and long-term follow-up remain difficult, limiting the certainty of current conclusions.

In the future, the development of a nationwide, linked individual-level database is essential. By integrating vaccination history, medical history, prior infection status, healthcare utilization, and mortality data, it will become possible to conduct analyses that support robust causal inference. Appropriate analytical approaches would include national cohort studies, self-controlled case series (SCCS), interrupted or time-series analyses with rigorous confounder adjustment, and broader causal inference frameworks applied to linked individual-level datasets. Key outcomes should be assessed separately, including all-cause mortality, excess mortality, COVID-19–related mortality, and major adverse events.

Several countries have already established nationwide systems linking person-level healthcare records with vaccination registries, enabling large-scale pharmacoepidemiologic studies with appropriate confounding adjustment (16). The UK Clinical Practice Research Datalink (CPRD), Denmark’s Danish National Patient Registry (DNPR), and Taiwan’s National Health Insurance Research Database (NHIRD) provide instructive models for nationwide individual-level linked health databases (29–31). Key lessons for Japan include centralized yet secure governance, robust privacy protection through standardized and anonymized data linkage, continuous data quality assurance, and longitudinal analytical frameworks linking routine care with hospitalization and mortality outcomes. Participation in international common data model–based research networks and the use of advanced methods for confounding adjustment may further strengthen the scientific utility and reliability of Japanese health data infrastructure.

In Japan, linked databases such as the Life Study (32), which integrates medical claims, long-term care claims, and health check-up data at the individual level, exist; however, these have been developed through researcher-led collaborations with individual municipalities rather than through a centrally coordinated national system. Municipalities already hold much of the data necessary to evaluate vaccine benefits and risks. Under the Next-Generation Medical Infrastructure framework, linkage of vaccination records with the National Database (NDB) and related systems is currently underway, with operational launch planned for 2026 (33). Nevertheless, database construction, governance frameworks, and analytical methodologies remain under active development. Accordingly, establishing a transparent nationwide data infrastructure together with robust analytical standards is a critical priority for appropriately evaluating vaccine effectiveness and safety in Japan.

## Discussion

At present, excess mortality in Japan remains elevated and is likely the result of multiple interacting factors, including population aging, changes in healthcare delivery, delayed access to medical care, and broader social determinants. It cannot be explained by a single cause, and no causal role of repeated booster vaccination can be inferred from the current evidence.

International data generally indicate that booster vaccination reduces COVID-19–related hospitalization and mortality, particularly among older and high-risk populations, although its effects on all-cause or excess mortality remain uncertain. In Japan, however, direct evaluation is limited by the absence of integrated person-level datasets linking vaccination history, clinical outcomes, and mortality.

Accordingly, current Japanese evidence—largely based on observational, ecological, and descriptive studies—remains insufficient for causal inference regarding repeated booster vaccination and excess mortality. Observed correlations, including associations between booster uptake and mortality trends, should be interpreted as exploratory signals rather than evidence of vaccine benefit or harm. While these studies provide useful descriptive signals, they are vulnerable to residual confounding and selection bias and should be interpreted accordingly.

The present discussion is therefore limited to identifying a potential “signal” associated with booster vaccination and should be regarded as hypothesis-generating in nature. Without integrated vaccination, clinical, prior infection, healthcare utilization, and mortality records, Japan currently lacks the infrastructure necessary to adequately resolve causal questions regarding repeated booster vaccination and excess mortality. The present uncertainty therefore reflects limitations in data integration and analytical capacity rather than definitive evidence either for or against a causal relationship.

Importantly, the absence of causal evidence from current Japanese studies does not preclude the possibility of a genuine causal relationship; rather, it reflects limitations in available data infrastructure and analytical capacity. Robust, individual-level analyses with appropriate confounding adjustment remain essential to definitively establish or refute such associations.

This limitation becomes clearer when contrasted with countries that maintain nationwide linked health databases. Systems such as the UK CPRD, Denmark’s DNPR and Taiwan’s NHIRD enable linkage of vaccination records, clinical information, and mortality outcomes at the individual level, thereby supporting large-scale pharmacoepidemiologic analyses with confounder adjustment and longitudinal follow-up. By comparison, Japan’s current fragmented data environment limits the certainty of causal conclusions.

Iwasaki recently wrote in her article (34): “PVS (post-vaccination syndrome) is a poorly characterized constellation of heterogeneous, non-specific symptoms that lacks an agreed-upon clinical definition, diagnostic criteria or standardized measurement. Owing to the stigma and judgement that surrounds this condition, scientists are unable to freely inquire about the risk of PVS without being labelled as ‘anti-vaxxers’.” She goes on to say that the goal should be “to protect the right to ask inconvenient questions while adhering to high scientific standards for evidence.” For that purpose, it is essential to establish an honest exchange of information backed by transparent data systems and rigorous confounder-adjusted analyses.

Given the exploratory nature of the currently available evidence, caution is warranted in interpreting any proposed relationship between COVID-19 vaccination and excess mortality. Public and scientific interest in this topic has been substantial, particularly in the context of persistent excess mortality observed in several countries. However, the existing evidence base remains limited, heterogeneous, and largely observational. Current findings should therefore be regarded primarily as hypothesis-generating rather than as evidence supporting or refuting a causal relationship. Future discussions should remain grounded in rigorous epidemiological methods and high-quality individual-level data rather than conclusions drawn from incomplete evidence.
