# Correction: Persistent excess mortality in post-pandemic Japan: current evidence, methodological limitations, and future directions

**DOI:** 10.3389/fpubh.2026.1930686

**Published:** 2026-07-23

**Authors:** Hiroshi Kusunoki, Ryota Sakai, Yumi Watanabe, Hideki Kakeya

**Affiliations:** 1 Department of Internal Medicine, Osaka Dental University, Hirakata, Japan; 2 Department of Rheumatology and Clinical Immunology, Saitama Medical Center, Saitama Medical University, Kawagoe, Japan; 3 Division of Preventive Medicine, Niigata University Graduate School of Medical and Dental Sciences, Niigata, Japan; 4 Institute of Systems and Information Engineering, University of Tsukuba, Tsukuba, Japan; 5 Transdisciplinary Review Association, Moriya, Japan

**Keywords:** all-cause mortality, booster vaccination, COVID-19, excess mortality, healthy vaccine bias, individual-level data infrastructure, methodological limitations

There was a mistake in the caption of Figure 2 as published. There was an error in one of the reference numbers.

The corrected caption of Figure 2 appears below:

“Unstandardized all-cause mortality rates by number of vaccine doses. Age groups: 20–49 **(A)**, 50–64 **(B)**, and 65–89 **(C)**. Error bars indicate 95% confidence intervals. The figures are adapted from Reference 16. Unstandardized all-cause mortality rates by number of vaccine doses, with a 90-day window after vaccination applied. Age groups: 20–49 **(D)**, 50–64 **(E)**, and 65–89 **(F)**. Error bars indicate 95% confidence intervals. The figures are adapted from Reference 17. Vaccination dose number was treated as a time-dependent covariate, and person-time was allocated dynamically according to changes in vaccination status. Mortality rate comparisons and confidence interval estimation were based on Poisson regression, which assumes equidispersion between the mean and variance. The study did not explicitly specify whether a post-vaccination lag or masking period (e.g., 14 days), during which individuals would remain classified under their previous dose category or be excluded from analysis, was implemented. Follow-up termination due to administrative censoring or relocation implicitly assumes non-informative right censoring, whereby censoring occurs independently of mortality risk. Furthermore, although analyses were stratified using broad age bands (e.g., 20–49 years), no within-stratum age standardization or adjustment was performed to address residual differences in age distribution.”

There was an error in the reference numbering in **Introduction**, *Development of nationwide data infrastructure for vaccine safety evaluation in Japan*, paragraph 3.

The corrected sentence appears below:

“Several countries have already established nationwide systems linking person-level healthcare records with vaccination registries, enabling large-scale pharmacoepidemiologic studies with appropriate confounding adjustment (16).”

The original article has been updated.

